# Code smells analysis for android applications and a solution for less battery consumption

**DOI:** 10.1038/s41598-024-67660-z

**Published:** 2024-07-26

**Authors:** Aakanshi Gupta, Bharti Suri, Deepanshu Sharma, Sanjay Misra, Luis Fernandez-Sanz

**Affiliations:** 1 Department of Computer Science and Engineering, Amity University Uttar Pradesh, Noida, India; 2 University School of Information, Communication, and Technology, Guru Gobind Singh Indraprastha University, New Delhi, India; 3 Computer Science and Engineering Department, Guru Gobind Singh Indraprastha University, New Delhi, India; 4 Department of Computer Science and Communication, Østfold University College, Halden, Norway; 5 Department of Applied Data Science, Institute for Energy Technology, Halden, Norway; 6 Department of Computer Science, University of Alcala, Alcala, Spain

**Keywords:** Android code smells, Software energy model, Green energy, Refactoring, Machine-learning, Robust statistics, Multi-linear regression, Computational science, Computer science, Information technology, Energy storage

## Abstract

In the digitization era, the battery consumption factor plays a vital role for the devices that operate Android software, expecting them to deliver high performance and good maintainability.The study aims to analyze the Android-specific code smells, their impact on battery consumption, and the formulation of a mathematical model concerning static code metrics hampered by the code smells. We studied the impact on battery consumption by three Android-specific code smells, namely: No Low Memory Resolver (NLMR), Slow Loop (SL) and Unclosed Closable, considering 4,165 classes of 16 Android applications. We used a rule-based classification method that aids the refactoring ideology. Subsequently, multi-linear regression (MLR) modeling is used to evaluate battery usage against the software metrics of smelly code instances. Moreover, it was possible to devise a correlation for the software metric influenced by battery consumption and rule-based classifiers. The outcome confirms that the refactoring of the considered code smells minimizes the battery consumption levels. The refactoring method accounts for an accuracy of 87.47% cumulatively. The applied MLR model has an R-square value of 0.76 for NLMR and 0.668 for SL, respectively. This study can guide the developers towards a complete package for the focused development life cycle of Android code, helping them minimize smartphone battery consumption and use the saved battery lives for other operations, contributing to the green energy revolution in mobile devices.

## Introduction

Smartphone users usually experience critical situations when their battery level is low. The primary cause of this is the mobile hardware, along with the kind and number of applications installed on the phone. So, it is crucial for developers to consider the quality of code and battery consumption by software during the software development life cycle and software maintenance life cycle. Such shortcomings in software quality may lead to adverse effects on the user experience of applications. It is also observed that when mobile applications are erroneously programmed, they can quickly drain device resources, such as memory, CPU, and energy, which results in low performance and software design defects that might hinder the maintainability of the software^20,35^.

The usage and consumption of phone batteries nowadays is a problem for people everywhere.^7^. The use of software is growing at the same rate as the global population. As a result of the increased uses of software in mobiles, energy consumption has increased considerably^2^. Every action that goes in creating the software contributes to the increase in software cost and carbon emissions. Green software engineering^20^, as its proponents refer to it, is a new paradigm aimed at producing green-enabled software with less negative environmental consequences. These software solutions are based on breakthroughs in research corresponding to the software development processes and the refactoring techniques.

While inspecting the quality of a software application, the design contributes significantly to software performance. Fowler, in 1999, devised the term code smells to indicate possible flaws in code design, violation of the design principles and best practices, affecting the maintenance and understanding, inflicting a negative impact on software quality^5,54^. Code smells tend to be the poor implementation of the design, leading to deviations from the expected execution that hampers the quality and maintainability of any software application^28^. Reiman et al.^29^ defined a set of Android developers’ bad programming practices as Android-specific code smells, which may threaten some non-functional attributes of mobile applications like source code quality, security, or data integrity. Additionally, he presented some refactoring techniques to eliminate those smells and enhance the code quality. In recent years, the number of Android applications has experienced a drastic increase from the user’s point of view as the usage of technically advanced Android smartphones grew rapidly. The software has also been shown to be the root cause of energy consumption in the latest study^64^. They limited their work by working in a controlled environment and performing refactoring manually.

The problem is especially apparent in the context of mobile phone applications when many users rely on smartphones for a range of activities. Recent advancements in this area include determining the influence of Android code smells on energy consumption and mobile application performance owing to poor programming practices^61^. However, the proposed tool^61^ can detect and fix only 5 Android code smells. Studies are conducted on the effect of code on energy optimization^24^. Tarwani et al.^75^ suggested applying the refactoring technique in order to achieve optimum software sustainability. They applied the refactoring techniques on the mobile applications. Since refactoring techniques remove bad smells, they help reduce energy consumption. A recently published study^73^ used three different refactoring strategies on source codes for mobile phones and computers and established a positive relationship between energy consumption and refactoring techniques. These aspects gave impetus for us to provide a mathematical model for analysing battery utilisation using software code metrics of smelly code, with the goal of preserving battery energy. The metric distribution that influences battery consumption and the metric distribution associated with refactoring rules have been established.

Ensuring the quality and effectiveness of code is crucial in the quickly changing world of Android app development. However, the maintainability, performance, and user experience of Android applications can all be adversely affected by the existence of code smells. Although code smells are becoming more widely acknowledged as a major problem in software development, there is still a dearth of thorough research and comprehension of the frequency, consequences, and Android-specific mitigating techniques of these issues.Consequently, this problem statement centres on carrying out an organised investigation of code smells unique to Android in order to respond to the four significant research questions:

### RQ1

“How can the software developers be helped in identifying the code smells as soon as possible?”

### RQ2

How much do Android code smells affect the amount of battery consumed by mobile applications?

### RQ3

Can a relationship for estimating battery usage through software code metrics of smelly code be derived?

### RQ4

How does the Android application’s battery consumption metric correlate with the metric associated with refactoring rules?

The RQ4 is to determine a relationship between the metrics that are reflected in the refactoring rules and in the mathematical model for evaluating battery usage.

Regarding the solution of these questions various machine learning algorithms with Feature Selector: Gain Ratio and Searching Approach: Ranker and statistical analysis like paired T-Test, Shapiro- Wilk a normality Test and robust statistics : Tukey Biweigh have been applied. Also, mathematical model have been developed for the relationship between battery usage through software code metrics of smelly code.

Due to the diverse field in which the Android operates, this study will be confined to Android-specific Java code smells as it is the most preferred language for Android-specific mobile application development^38^. Most of the published^11,22^ research work is dedicated to Android smells ranging from 2014 to 2019^9,14^. It implies that Android smells are still at their emergence stage and need additional contributions to improve results. Subsequently, the detection and refactoring of smells in Android have become a budding domain for researchers. This work explores the Android-specific code smells and their impact on battery consumption and the subsequent performance issues. The code smells were manually refactored and tested along with the smelly code to explore various quantitative measures.

The practical implementation of the research work contributes to the following findings:

- To analyze the impact of Android code smells on battery consumption to improve energy efficiency.
- To quantitatively analyze the difference in metric distribution and the composition of the changes in percentage observed before and after refactoring.
- To develop a mathematical model for evaluating battery usage through software code metrics of smelly code, promoting battery energy preservation.
- To devise a correlation between the metric distribution affecting the battery consumption and metric distribution associated with refactoring rules.

As per our knowledge, in the available literature Palomba et al^24^ research enhanced comprehension of energy consumption components of Android application development and provided empirically-supported recommendations for mitigating energy leakage via code refactoring techniques. It is possible that Pereira et al.^73^ research provided important insights into the intricate interactions between variables affecting Android device battery life, guiding the creation of more energy-efficient hardware and software solutions and enabling users to take control of their device’s battery life. However, the presented study have drafted a mathematical formula linking battery change and static code metrics. Again, none of them has devised a correlation between smelly code and refactored software metrics. These results would help developers produce smell-free source code and reduce the battery consumption of Android applications, leading to sustainable and maintainable software applications.

Structure of the Paper: The paper is structured as follows: In “Background study” section presents the study of the background and related works. In “Research study and design” section illustrates the research study definition and design. In “Result and discussion” section discusses the result and implications of the performed work and “Threats to validity” section analyses the threats to validity. Finally, “Conclusion and future scope” section presents the conclusions and depicts the future scope of the research.

## Background study

This section reviews the literature to analyze the published contributions relevant to code smell detection techniques and the effects of code smell on battery consumption in software applications.

### Evolution of code smell and refactoring

Fowler et al. described the concept of code smells as design anomalies^24^. Code smells appear when the design rules of the software vary from the optimal pre-defined conventional design rules. Code smells have been discussed for languages like Java, C++, Python, Ruby, and Scala^21,33^. Fowler proposed 22 generic code smells which are language-independent. Systematic literature^8^ already reviewed a list of detection tools such as ’infusion’, ’JDeodorant’, ’iPlasma’, and ’PMD’. As per our research, the first broad evaluation of several detection tools and code smells was published in^4^. Going further from Fowler’s generic smells, Reimann et al.^35^ presented a catalog of thirty Android-specific code smells relative to both implementation and user interface designs, which mainly targeted Android-based mobile applications. Automated Android smell detection also started with two tools: ’PAPRIKA’ and ’aDoctor’. PAPRIKA^11^ identifies Android-specific and object-oriented code smells using Android application package (APK) files. The other tool ’aDoctor’, is publicly available for usage and supports both command-line interface (CLI) and graphical user interface (GUI) developed by Palomba, which identify 15 out of 30 Android code smells^23,28^. The aDoctor tool is based on the class-level granularity, which categorizes the smell based on its absence and presence.

The identification of code smells significantly impacts the performance and quality of the software application^11,16^. Tufano et al. stated the reasons for code smells and their survivability^36^ after studying the developer’s contributions during the evolution of a software application that favor the inclusion of Android code smells^10^. Apart from the detection techniques, practitioners have implemented many refactoring methodologies to reduce the risk of performance degradation. One option is automated refactoring tools such as Leafactor developed by Cruz et al.^46^. Another refactoring tool, ’EARMO’ refactors Android-specific code smells in mobile applications^26,50^. Some practitioners prefer to apply manual refactoring techniques depending on time and human resources^38^. Further studies on code refactoring have identified the code patterns involved in massive energy consumption and removed them from source code^43,52^. Verdecchia et al.^69^ established an organised set of rules for developers to follow in order to improve the design of Android apps.

### Code smells impact on energy consumption

In terms of energy consumption, one way to reduce smartphone energy consumption is to improve software quality. Software should consist of effective, low-resource-consuming, and, in particular, code smell-free programming.

Palomba et al.^24^ discussed the results of a recent empirical investigation that looked at the impact of nine Android-specific code smells on mobile app energy consumption. They investigated whether refactoring processes could help with energy leakage reduction. However, the work^24^ highlighted about the 4 Android code smells. There is also an absence of an automatic refactoring tool. Kim et al.^67^ identified energy-consuming constructions as suspicious codes that are expected to cost a significant amount of energy and then developed strategies to remove them. Carette et al.^42^ introduced HOT-PEPPER, a tool-based and reproducible approach for automatically correcting code smells and assessing their influence on energy consumption. They have not considered the pictures’ smell, which are based on samples. Additionally, they have not considered the energy mentioned by non-intrusive methods. Anwar et al.^65^ examined the influence of numerous typical code refactoring in Android applications on performance and energy consumption. According to the findings of the experiments, various code smell refactoring has a significant impact on the energy consumption of Android apps. They have limited their work to only three applications. As a result, there is a lack of generality of the findings. Rodríguez et al.^68^ found that removing bad smells increases battery consumption in mobile apps while ensuring crucial features of object-oriented design, such as maintainability and flexibility. Verdecchia et al.^60^ demonstrated that refactoring code smells can result in considerable reductions in software application energy usage.

According to the findings of Hecht et al.,^11^, fixing Android code smells improved UI and memory performance. When the three Android code smells were corrected, there was a 12.4 percent increase in UI metrics and a 3.6 percent improvement in memory-related metrics. Dhaka et al.^44^ empirically investigated the impact of eliminating a collection of three prominent code smells, individually and in all six conceivable sequences, on the energy consumption behaviour of software systems. Lee et al.^66^ offered refactoring methods for reducing energy consumption and enabling software development and maintenance to meet energy requirements. They’ve also defined energy bad smells, such as code patterns that consume a lot of energy and refactoring strategies to get rid of them. Refactorings, according to Sahin et al.^70^, can not only influence energy utilisation but also raise or decrease the amount of energy needed by an application. For a group of mobile apps from the Google Play store, the Hao et al.^71^ technique can estimate energy consumption to within 10% of the ground truth. Green Miner is the first dedicated hardware mining software repositories test-bed, according to Hindle et al.^72^. The Green Miner is a gadget that physically detects the energy usage of mobile devices (Android phones) and automates application testing and reporting to developers and academicians. Pereira et al.^73^ presented their research in order to better understand how Android apps, operating systems, hardware, and user habits affect battery life. Pinto et al.^74^ discovered a link between design decision options and settings and parallel systems’ energy consumption.

### Energy measurement method

According to Saborido et al.,^47^, the voltmeter used in their experiment to estimate energy consumption operates at a frequency of only 10 Hz, which is too low to see actual method usage, potentially leading to erroneous results. Invoking methods, accessing fields and modifying the length of arrays were all investigated in a small-scale empirical study (four code snippets) by Li et al .^48^. The findings of this investigation corroborated the predictions, suggesting that such strategies can help minimise the amount of energy used by mobile apps. In comparison to the original colour palette, Mario Linares et al.^49^ proposed GEMMA (Gui Energy Multi-objective optimization for Android apps), a multi-objective optimization technique for generating colour palettes that produced colour solutions that optimised energy consumption and contrast while using consistent colours. GEMMA created solutions that reduce energy use while maintaining consumer acceptance.

Morales et al.^50^ presented EARMO, a refactoring tool that considers energy consumption in addition to code quality when addressing code smells in mobile apps. The study’s outcome, on the other hand, is device-specific.

For assessing the energy profile of mobile apps, Nucci et al.^51^ proposed a software-based tool ‘PETRA’. It was put to the test with 54 apps and the estimation error was found to be less than 5% of the real values collected with a hardware-based tool. Verdecchia et al.^60^ demonstrated that refactoring code smells could result in considerable reductions in software application energy usage in addition to the existing research on the benefits of code maintainability. The expanded version of the ’aDoctor’ tool was presented by Iannone et al.^61^ to discover and refactor five Android-specific energy smells.

Ribeiro et al.^62^ developed EcoAndroid, an Android Studio plugin that automatically adds energy patterns to Java source code. EcoAndroid was able to automatically refactor all the code smells detected. Cruz et al.^46^ investigated eight strategies for lowering the amount of energy consumed by Android apps. They discovered that adding energy-aware approaches on six popular apps could extend the smartphone’s battery life. Palomba et al.^24^ presented the analysis of a recent empirical study on the influence of nine Android-specific code smells on mobile app energy usage. They investigated if refactoring operations could support in the reduction of energy leakage.

### Code smell analysis through machine learning

The apriori algorithm was used in the study by Palomba^25^ to generate association rules for co-occurrences of code smells. Fu et al.^6^ used it to study the evolution of different versions of a system. Khomh et al.^15^ adopted a Bayesian approach to study code smells. Gupta et al.^9^ study focused on detecting Android-specific code smells using a machine learning classifier model with detection rules. Gupta et al.^77^ proposed a code smell prediction model using entropy’s concept. An additional aspect studied by the research community has been analyzing the impact of code smells on maintenance activities. Lozano et al.^18^ proposed an approach to change history information to better understand the relationship between code smells and the violations of design principles to assess the cause of code smells. They have also highlighted the influence these smells have on how developers apply the inheritance mechanism. Gupta et al.^77^ presented a systematic literature review on Java code smells in which they listed various code smells with their respective detection machine learning algorithms.

The study^80,84^ used a hybrid Fuzzy ANP-TOPSIS approach, which gave designers and developers an equitable way to improve software security by rating security features and setting priorities for approaches. This article^81^ improved the usability of reliability prediction models, assisting developers in lowering failure rates and enhancing software reliability by examining prior work and making recommendations for enhancements. In this study^82^ developed a fuzzy logic and neural networks, the paper tackles the problem of nonlinear parameter estimation in software reliability methods. This research showed promising results on large datasets from Apache server and MyLyn application software . The study^83^ presented a hesitant fuzzy set systems to handle uncertainty and presents hesitant fuzzy multi-factor decision analysis strategies to choose the best renewable energy sources. The results showed that biogas and landfill gases are the best options, outperforming other techniques in terms of accuracy. Overall limited work focuses on minimizing the Android code smells in the literature.

The paper^85,86^ highlighted the growing need for long-lasting security and introduced the importance of security in software engineering. In order to improve security attributes like dependability, trustworthiness, and human trust, it suggested a prioritising strategy using the Fuzzy Analytic Hierarchy Process (Fuzzy AHP). The research^87^ emphasised the difficulty in choosing appropriate analysis methods as well as the frequency of malware in cyber attacks. This study compared various techniques for analysing malware in web applications using Fuzzy TOPSIS and Fuzzy AHP, and analyzed that Reverse Engineering is the most efficient method.

**Table 1:** Related papers which have the keywords: Android code smells+ Energy + Mobile applications.

S.No.	Author	Title	Methodology	Results/implications	Limitations
1	Palomba et al.	On the impact of code smells on the energy consumption of mobile applications.	The authors conducted a large-scale empirical study on the influence of 9 Android-specific code smells on the energy consumption of 60 Android apps. For code smell detection, they have used the aDoctor tool and for energy estimation, the ’PETRA’ tool has been used.	They have concluded that the following are the most frequent smells: Leaking Thread, Member Ignoring Method, Slow Loop, and Data Transmission Without Compression, and also that Refactoring smelly code is a crucial task for increasing energy efficiency.	Automatic refactoring tool is not mentioned. This work conducted a empirical study.
2	Carette et al.	Investigating the energy impact of Android smells.	Two key tools, PAPRIKA and NAGA VIPER, complement the HOT-PEPPER technique. When calculating the energy metrics for each APK file, HOT-PEPPER makes use of NAGA VIPER and PAPRIKA to identify Android code smells. The most energy-efficient APK, the accompanying source code, and a list of refactorings from Paprika’s rectification are returned by NAGA VIPER after comparing these energy measures.	When the three code smells (Internal Getter/Setter, Member Ignoring Method, and HashMap Usage) are fixed, they have observed a worldwide decrease in energy consumption in one programme by 4.83 %.	They did not consider the pictures’ smells which are based on samples. Additionally, they did not consider energy mentioned by non-intrusive methods.
3	Morales et al.	Earmo: An energy-aware refactoring approach for mobile apps.	They have offered a strategy where they have created refactoring sequences for EARMO (Energy-Aware Refactoring Approach for Mobile Apps), which is based on a search-based method to enhance the design of an app. This procedure entails analysing a number of iterative refactoring sequences as well as the final design in terms of design quality and energy usage.	In less than a minute, EARMO can produce refactoring suggestions and eliminate, on average, 84 percent of anti-patterns.	The result of the study is specifically device-dependent.
4	Anwar et al.	Evaluating the impact of code smell refactoring on the energy consumption of Android applications.	Espresso tool is used to verify the accuracy of the refactored code. The Monsoon power monitor was then used to measure energy usage.	The results showed that the largest energy savings for refactoring the code smells “Duplicated code” and “Type Checking” were 10.8% and 10.5 percent, respectively.	They have limited their work to only 3 applications.
5	Hecht et al.	An Empirical Study of the Performance Impacts of Android Code Smells	An empirical analysis concentrating on the effects of three Android performance code smells on two open source projects is presented in this paper. The frame time, number of delayed frames, memory usage, and number of garbage collection calls were used to assess the UI (User Interface) and memory performance.	The outcomes demonstrate that fixing these Android code smells significantly enhances UI and memory performance. When the Member Ignoring Method is corrected, we specifically see improvements of up to 12.4% in UI metrics and up to 3.6% in memory-related metrics.	Only one code smell was fixed. The majority of the tests they have used are non-parametric ones, which do not call for assuming anything about the distribution of the metrics.
6	Moreira et al.	Overviewing the Liveness of Refactoring for Energy Efficiency	The available lead factor tool is used to examine real-time feedback regarding a program’s energy efficiency as it is being programmed.	The code smell ’findviewbyid’ causes a 4.5 percent increase in energy usage. They found 13 different tools and split them into two groups: tools to measure energy use and tools to improve energy usage.	Leafactor’s liveness rating is barely 3. Leafactor only supports the Eclipse IDE, which is not the most popular for developing Android applications.
7	Ribeiro et al.	EcoAndroid: An Android Studio Plugin for Developing Energy-Efficient Java Mobile Applications	The source code was refactored with the help of the IntelliJ Program Structure Interface (PSI). Its features also allow for the discovery of prospective energy improvements.	An Android Studio plugin called EcoAndroid is proposed, which automatically applies a number of energy-saving techniques into Java source code.	The proposed EcoAndroid is a complex system, so there could be bugs in the implementation.
8	Cruz et al.	Using Automatic Refactoring to Improve Energy Efficiency of Android Apps	Lefactor tool is applied for code smell detection and 140 open source Android apps are considered	Five energy code smells are identified using Lefactor tool. The refactoring changes were successfully merged into 40% of the apps.	Only five energy refactorings have been taken into account.
9	Şanlıalp	Energy Efficiency Analysis of Code Refactoring Techniques for Green and Sustainable Software in Portable Devices	The energy usage was calculated using Trepn Profiler and Intel Power Gadget, two software power estimators.	The suggested method has been successfully applied to 2048 datasets for desktop and mobile applications, demonstrating its superiority in refactoring technique combination prediction.	They have dealt with experimentation in a controlled environment.

In Table^1^, we have presented the previous studies that have a similar goal. They are likewise examining and analysing the effect of Android code smells on battery usage for mobile applications. Our research is a parallel exploration from a different perspective. A mathematical model has been proposed in this research study to ensure that the results will be more accurate. As a result, it’s conceivable that some code smells affect energy consumption in a way that isn’t clear from just one evaluation.

It’s worth mentioning that our study aims to better understand the impact of a variety of Android-specific code smells. The identified contributions have tried to understand the effects of Android-specific code smells harming the battery life and the role of refactoring in improving the code base. The aim of our work is to suggest a model that increases the efficiency of the applications and focuses on the battery consumption of Android applications, resulting in sustainable software development. The goal is to identify a relationship between the code metrics and battery usage, thus enabling a clear understanding of code smells’ implications on power and energy. Table 1 shows the methodology, result/implication, and limitations of the related studies.

## Research study and design

### Methodology

The study’s purpose is to create a mathematical model for measuring battery utilisation using smelly code metrics in order to promote battery energy conservation. The goal is to establish a link between the metric distribution that affects battery usage and the metric distribution that affects refactoring rules. As a result, we’ve deduced the four research questions. The following are the research questions that we are going to investigate:

#### RQ1

“How can the software developers be helped in identifying the code smells as soon as possible?”

Typically, code smells are discovered during the maintenance phase of the SDLC (Software Development Life Cycle), and the refactoring process is used to eliminate them. Since refactoring is a very time-consuming process, having software metrics-based rules can help improve software quality. The extracted rules can help the developers to enhance the code quality while the software is under development.

Therefore, machine learning algorithms are applied to the metrics, which will compute the human-readable rules for identifying the code smells as soon as possible.

#### RQ2

How much do Android code smells affect the amount of battery consumed by mobile applications?

The RQ2 is formed to understand the relationship between battery consumption and Android code smells in mobile applications. We evaluate the smelly mobile software and the refactored software that leads to finding the impact of battery usage in the considered smelly applications.

#### RQ3

Can a relationship for estimating battery usage through software code metrics of smelly code be derived?

RQ3 is for finding a relationship between the battery consumption and the smelly code metrics reflecting changes after refactoring. Afterward, a mathematical model is derived for battery consumption.

#### RQ4

How does the Android application’s battery consumption metric correlate with the metric associated with refactoring rules?

The RQ4 is to determine a relationship between the metrics that are reflected in the refactoring rules and in the mathematical model for evaluating battery usage.

Thus establishing a correlation between battery usage and software code metrics.

**Figure 1:**
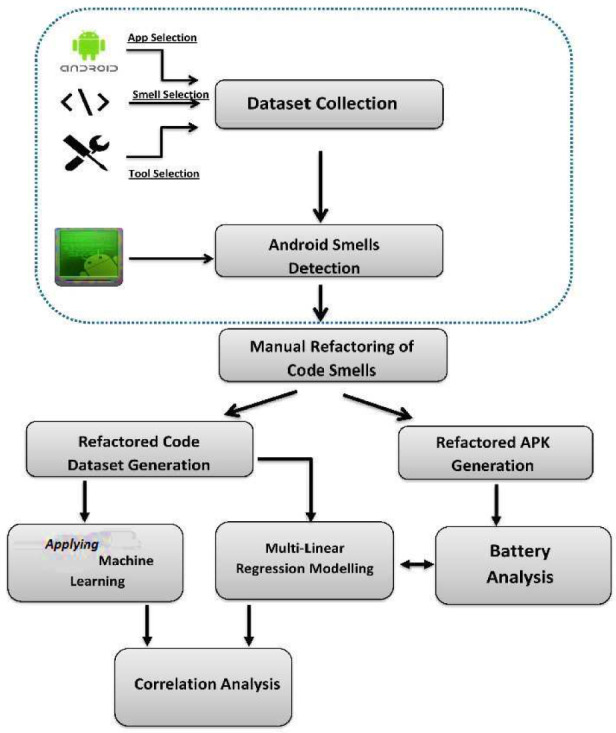
Workflow of the research study.

Figure 1 describes the proposed approach for the research study. The preliminary step is the selection of Android applications from open-source repositories. The source code files have been analyzed to find Android-specific code smells^23^. We manually refactored them to eliminate the code smells in all applications, as the target classes with smells were detected by the smell detector. We collected static code metrics (https://www.meteonic.com/understand) for the smelly and the refactoring source code after performing the refactoring process. For the rest of this paper, the refactored code of the applications will be referred to as a refactored version, whereas the smelly code will be referred to as a smelly version.

Further, we applied machine learning algorithms, evaluating the best algorithm among all to formulate the refactoring rules. A manual analysis of the battery consumption for both smelly and refactored source code using the Android debug bridge (ADB)^76^ was the basis for determining the impact of refactoring over the smelly code. The ADB (Android Debug Bridge) is a command-line tool developed by Google to facilitate communication between computer systems and Android devices^76^. ADB helped to reset the battery state using Unix shell commands in Android device and then connecting to RQ2.

We recorded both the code smells hampering the applications’ battery consumption and the metrics involved in refactoring replica rules to establish a correlation among them. Eventually, we analyzed the calculated percentage of battery consumption for the code smell and the metrics affected by it with a multi-linear regression model.

### Context selection

The context of this study is focused on the Android platform, specifically aiming at Java-based Android applications. This section shows how the application selection, the Android code smell selection, and its detection were carried out.

#### Application selection

We based our study on open-source Android applications with Java programming language extracted from a global repository in GitHub. We cloned 16 software systems from the open-source repositories, totaling 4,165 classes and 12,764 methods. The main criteria for systems selection are its occurrences and reputation on the GitHub platform, which is already proved by popular applications. These software are the most popular, most used, and most trusted by the user, thus making them a suitable choice for research study. In addition, we prioritized applications exhibiting basic utility-based functionalities to target many Android users and their daily mobile software requirements. We prioritized applications exhibiting basic utility-based functionalities to target many Android users and their daily mobile software requirements. So, we have mainly considered code smell analysis applications such as login systems, sound recording, messaging (SMS), podcasting, planning, and social media in this study. The entire dataset of 16 applications contains 730 manually validated smelly instances for three considered code smells. Each code smell is operated upon ten applications. This work is developed at the class level, and the selected applications are listed in Table 2.

**Table 2:** Name and description of the applications considered in this research.

Apps name	Description	# Classes	# Files	#Packages
AntennaPod	Open-source podcast manager.	908	546	138
AudioRecorder	Open-source sound recorder application.	416	71	18
ClipStack	A tiny clipboard history manager app	137	23	3
Group05	Route map designer app	35	21	6
LoginDemo	login system module app	12	19	3
MinimalTo-Do	Open source to-do-list application	151	200	35
AmazeMod	Material design file manager for Android	270	300	80
OmniNotes	Notes application for Android	783	853	143
ResturantBot	Resturant chat bot application	104	209	15
SoundRecorder	Sound recorder application for Android	39	52	7
StudentForum	Student forum application for blogfeeds	156	121	3
Termux	Android terminal, Linux env. based app	110	133	14
Timber	Material design music player	728	473	94
To-Do-List	To-Do-List application	32	42	3
Ucrop	Image cropping library for Android	225	298	33
UKVanhi	Social group application	59	62	14
		4165	3423	609

#### Code smell selection

In the academic literature, the main reference to Android code smells is the catalog of Reimann et al.^29^ containing 30 code smells specific to Android, which is mainly performance-oriented and covers various aspects like user interface and data. However, this study undertakes three code smells, namely NLMR, SL, and UC, which, according to their definition^29^, might get ignored by the developer, causing degradation in software quality. Moreover, these code smells in the analyzed applications play a vital role in the selection criteria. The criteria for selecting these three code smells is their frequent occurrences. And hence having a high impact on the application’s performance. The selected code smells are investigated at the class level. In this regard, the detection relies on the already mentioned aDoctor tool 1.0 (https://github.com/google/fpalomba/aDoctor). The tool helps in the detection of 15 Reimann-proposed Java-based Android-specific code smells^29^. The three Android code smells considered are defined in the Reimann catalog as follows:

- NLMR (No Low Memory Resolver): Mobile systems include a limited amount of RAM and less Virtual memory usage for space swapping. The ’onLowMemory()’ method in Android applications is used to kill processes in order to reclaim the part of memory. The absence of this overridable method terminates the processes to rescue any part of the memory in Android apps, and it is an indicator of a bad implementation of the design. The mobile properties like memory, efficiency, user experience, and stability are adversely impacted by NLMR code smell^29^.
- SL (Slow Loop): The conventional for loop is slow and expensive; hence an advanced version of it, ’Enhanced For Loop’, should be preferred in Android applications. It is believed that a hand-written counted loop is about 3× faster (with or without JIT) with an array list. However, for other data collections, the enhanced for-loop syntax will be exactly equivalent to the explicit iterator usage^29^.
- UC (Unclosed Closable): An object implementing the closable interface, if not closed, results in large memory consumption. Missing the close() method on objects implementing the closable interface is a bad practice^29^.

The detection of Android code smells relies on the ’aDoctor’ tool version 1.0. The indicated tool marks instances at the class level with Boolean figures help (0- the absence of code smell;1- the presence of code smell). The tool guarantees the developers a 100% precision, a 100% recall, and a 100% F-measure for the detection of considered smells by ’aDoctor’^29^.

#### Refactoring of smells

Refactoring is the process of improving software’s inherent properties by altering the external structure while preserving the internal logic. Martin Fowler^19^ was the first to write a book on the topic, with 70 refactoring tips and tricks. Refactoring contributes to constraining the complexity of source code and reveals a positive impact on the software quality^18^. Frequently refactored code is expected to be understandable, accurate, correct, and coherent in new environments. We implemented manual refactoring to eliminate the code smells by building and running the code on Android Studio version 3.5.3. The application’s nature confirmed the evaluation of successful refactoring through seamless deployment on both emulator and Android smartphones. Once the refactored code has been tested on Android studio, we validated again using aDoctor to detect any smell that could have been mistakenly left.

### Battery analysis

This section explains the different parameters and techniques used to collect and analyze the application data and their battery usage.

**Pre-requisites Setup** We set up the battery analysis environment that required Android Studio with proper Androids SDK and JDK versions. It is vital to use the latest SDK versions, so the stable release of Android SDK 26 following JRE 1.8.0 was preferred. Once the setup was completed, we analyzed all the applications used in this project with Android Studio, firstly for the smelly version and then for the refactored versions. After successfully building the code without any compile time or run time error, we analyzed the Android Application Package (APK) for running phones. We applied the battery consumption analysis on the real-time devices to ensure a realistic scenario that the user faces, so the APK was transferred to the phones for testing purposes. The phones included different battery functions, which helped in effectively measuring the changes in battery load percentage. All the phones showed changes while testing the smelly versus the refactored application. The configurations of smartphones were the following ones:

1. Xiaomi Redmi Note 5 Pro (Android 9KQ1.1) – 4000mAh.
2. Xiaomi Redmi Note 5 (Android 8.1) – 4000mAh.
3. Samsung M30S (Android 9) – 6000mAh.

We have also compared the battery percentage change value for both smelly and refactored versions of each application.

#### Battery usage evaluation

As discussed in the ’pre-requisites setup’ section, we successfully generated the APKs for both versions (smelly and refactored). These APKs run for a specific time period. It is worth mentioning that the actions performed for both versions of each application were almost the same, ensuring accuracy for the comparison of changes. Before battery analysis, it is crucial to install Android Debug Bridge (ADB) to communicate with the device through the command shell. However, it is not possible to just run the application and control the battery usage because many other applications run simultaneously, making it challenging to analyze the individual impact of applications on batteries. The inbuilt software of Batterystats in Android smartphones limits the percentage of battery usage to only one decimal unit and sometimes reflects changes for more extended running applications only. Therefore, we needed to use a tool named Battery Historian (https://github.com/google/battery-historian)^40^ to check the battery usage by each version of all the applications for respective smells.

**Figure 2:**
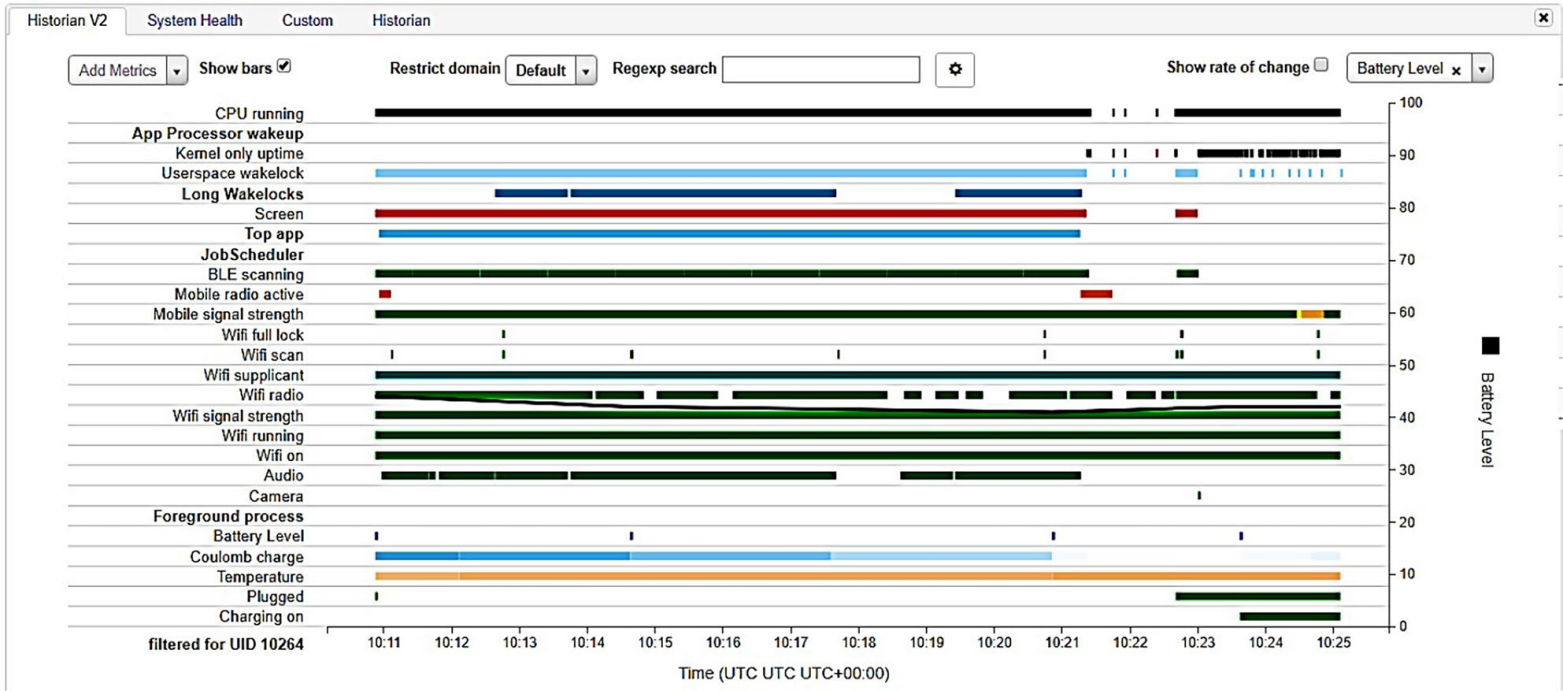
Graph of device resources’ battery usage.

Unlike the previously mentioned research carried out by Palomba (who automated the process and evaluated the results at class level^23^) our next step was evaluating the battery usage at the application level using Battery Historian by accepting the bug report text file as input and then converts the report analyzed from Battery stats into an HTML visualization, as shown in Fig. 2.

#### Bug report generation

During manual tests of both versions of applications (smelly and refactored), the battery stats feature of the Android operating system keeps track of battery capacity both at the system and application levels. In this research, we examined the battery usage for the two versions of each application (smelly and refactored) at the package level. The analysis uses ADB commands to reset the battery profile so that the battery stats do not consider the system’s prior data and start when the command is executed. The ADB reset command for battery profile in battery stats is the following one:

*ADB shell dumpsys batterystats –reset*

After resetting the battery profile, we initially analyzed the two versions of applications for 5 minutes. The process generated bug reports for each version of each application after the successful completion of battery computation. This bug report is shaped as a zip file with a bug report text document that contains all the battery stats information.

### Ethics approval

No ethical approval is required based on: a. This article does not contain any studies with animals performed by any of the authors. b. This article does not contain any studies with human participants or animals performed by any of the authors.

## Result and discussion

As the research progressed, we examined various new findings and relationships for the considered Android code smells. The presentation of research results is arranged according to the research questions mentioned in section “Methodology” .

### RQ1: How can the software developers be helped in identifying the code smells at the earliest?

The software developers can help in identifying the code smells by using the methodology followed by the author. The considered code smells, and their respective metrics dataset was extracted and then refactored. Afterward, the boolean identification is performed where the refactored class was labeled as “refactored True,” and the non-smelly class was labeled as “False”. Then, we collected corresponding static software metrics and prepared the dataset of refactored true vs non-smelly class. Subsequently, feature selection techniques were used to extract the important feature metrics, and the refined dataset was input to the considered algorithms for classification. Then, the paired-T test was used to compare different classifiers to extract the best algorithm for rule generation. In the last, we have calculated the best algorithm’s performance metrics and generated the software metric-based rules that can help the developers for developing efficient source code.

The in-depth process followed to answer this research question is done sequentially, as described below.

We extracted the data from 4,165 classes referred to the three smells (NLMR, SL, UC) using the ’aDoctor’ tool. A code walk-through technique was applied to verify the spread of code smells. The smells were removed through manual refactoring. This includes refactoring the smelly chunk of code so that internal functionality does not get compromised. This also implies that only the external structure gets altered.

**Figure 3:**
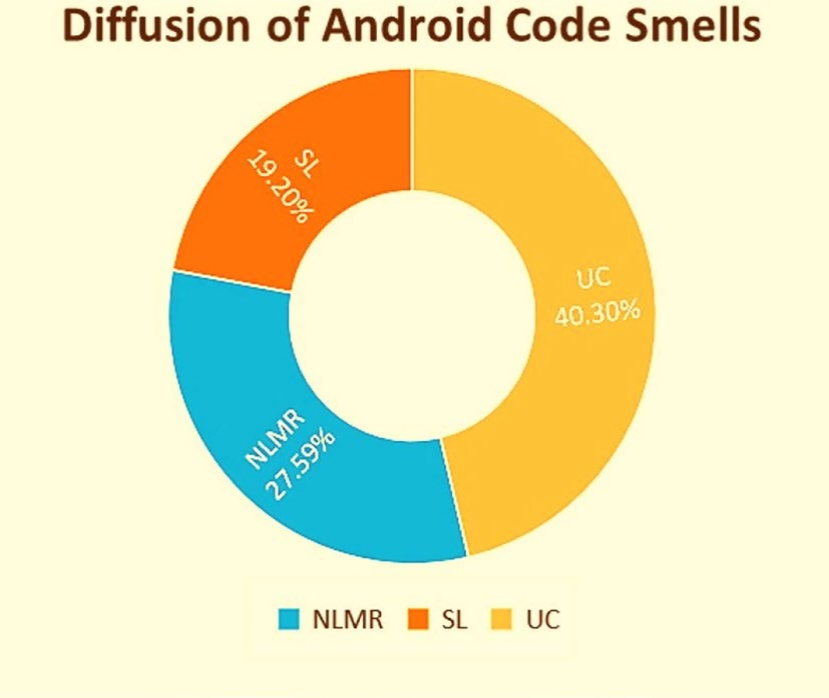
Presence of code smells in the Android applications.

We applied Reimann’s refactoring methods to eliminate the smells at the class level. It is relevant to note that every smell has been independently refactored. The total number of smelly classes for each smell (NLMR, SL, and UC) were 231, 161, and 338, respectively (see Fig. 3).

The next step was the analysis of code using a static code metric analyzer tool to extract the metrics. The software metrics are critical for measuring processes and products, such as extensibility, accuracy, flexibility, complexity, and other features. They are useful for estimating the quality of Android and object-oriented applications^13,34^. We used a static code analyzer tool, sciTools ’Understand (https://scitools.com/), to extract metrics at class and package level for Java programming language. The static metrics act as a source of authentication for predicting and anticipating the enhancement of source code. We extracted the metrics before and after refactoring the code. The classes that were identified as smelly and later refactored were marked with ’refactored true’, and others classes were marked as ’False.’ In this way, the labeled dataset has been prepared.

The next step was the machine learning analysis, as explained in the following subsection.

#### Machine learning approach

Once the labeled data set was ready, the next step was to identify the most significant software metrics from the 43 metrics obtained for further evaluation. Feature selection techniques minimize the dimension of the data by selecting a subset of vital features. The features are chosen to preserve complete information. The feature selection techniques used in this work are Feature Selector: Gain Ratio and Searching Approach: Ranker.

We then applied the corrected Paired-T test^12^ to examine the accuracy for various machine learning rule-based classifiers (Naive Bayes, JRip, ZeroR, OneR)^4^ using weka experimenter that is available in weka data-mining tool. Table 3 shows the detailed examination of classifiers with their accuracy as used in the study.

**Table 3:** Comparison of classifiers through paired-T test.

Paired-T test results				
Dataset	ZeroR	JRip	OneR	NaiveBayes
NLMR	79.86	81.06	79.56	46.09
SL	88.69	89.09	89.32	79.98
UC	92.41	92.23	92.15	51.88

According to the outcome, we could observe that the JRip rule-based classifier algorithm achieved the highest accuracy score measure. Subsequently, the machine learning rule-based classifier JRip was validated over 10-fold cross-validation to obtain the rules^4^. Then, we approximated the JRip algorithm’s effectiveness through various performance measures. These performance metrics, as shown in Table 4, help to measure the extent of the correctness of the classifiers on the given dataset. The performance metrics listed in the Table 4 will help to justify the effectiveness of the classifier model^4^:

**Table 4:** Performance measures of JRip algorithm.

Bad smells	TP rate	FP rate	Precision	Recall	F-Measure	Kappa statistics	Accuracy
NLMR	0.938	0.615	0.858	0.938	0.896	3[6]*0.3746	3[6]*0.826
	0.385	0.062	0.61	0.385	0.472		
	0.827	0.503	0.808	0.827	0.811		
SL	0.984	0.839	0.902	0.984	0.941	3[6]*0.2116	3[6]*0.891
	0.161	0.016	0.565	0.161	0.251		
	0.891	0.745	0.864	0.891	0.863		
UC	0.99	0.858	0.934	0.99	0.961	4[8]*0.1987	4[8]*0.925
	0.142	0.01	0.533	0.142	0.224		
	0.925	0.794	0.903	0.925	0.905		

The metric-based rules determined using the JRip algorithm would help the software developers for code smell detection. The rules in terms of software metrics for bad smell detection are given below:

- NLMR: (MaxInheritanceTree $>=$ 2) and (PercentLackOfCohesion $>= 66$) and CountStmtExe $>= 49$)
- SL: (CountStmtExe $>= 84$) and (CountLine $<= 565$)
- UC: (MaxInheritanceTree $>= 2$) and (CountLineCodeDecl $<= 9$) and (CountStmtDecl $>= 3$) and (SumCyclomaticModified $<= 2$) and (CountStmt $>= 17$)

Hence, RQ1 acknowledges a method that would help the developers identify the code smells as early as possible using the software source code metric threshold values, which improves the software quality of code and significantly impacts the software development life-cycle.

### RQ2: How much do Android code smells affect the amount of battery consumed by mobile applications?

Frequent battery consumption is an unpleasant experience for smartphone users. One of the reasons is the existence of code smells. We tried to analyze the change in battery consumption for real-life applications by eliminating the code smells to address RQ2.

An environment is set up to evaluate the battery consumption of refactored and smelly applications using the Android SDK and its toolsets. Then, we performed the battery consumption test manually (like opening the app, swiping left and right, login and logout, scrolling up and down), which included the basic test runs that are performed by the users while using an application. The reason to opt for the manual approach is to authenticate the results by replicating the human interaction in the real environment. Subsequently, the Shapiro–Wilk test of normality was applied to their distribution. Finally, a maximum-likelihood estimation was performed based on observed data on battery consumption, and the preserved battery percentage value was recorded.

**Bug Report Analysis:** The battery analysis for every application before and after refactoring the code smells is performed, as explained in section “Methodology”. Afterward, the results reflect the changes between both versions of the same application, with similar actions expressed in the form of battery usage percentage. However, when analyzing results from the 5-minute analysis, as explained in section “Methodology”, we observed really little relevant change between smelly and refactored versions of an application for the three smells. The same analysis was again performed for 10 minutes duration instead of 5 minutes.

**Test of Normality: Shapiro Wilk Test** The records are linked to the package level data for the two versions of each application, and the same process was repeated for each smell (NLMR, SL, and UC). However, the data obtained includes manual errors, too. We performed a Test of Normality^16^ with the data obtained from battery stats and package level metrics based on the two-tailed Shapiro Wilk Test^27^.

**Table 5:** Test of normality.

Shapiro–Wilk			
Battery change	Statistic	df	Sig.
NLMR	0.910	10	0.282
SL	0.917	10	0.330
UC	0.849	10	0.057

**Shapiro–Wilk Test:-** This test assesses the following hypothesis:

**Null Hypothesis (H0)—The data sample belongs to a normal distribution.**

**Alternative Hypothesis (Ha)—The data sample doesn’t belong to a normal distribution.**

A significant factor decides whether the applied tests follow the null hypothesis or the alternate hypothesis. For S-W tests, p-value > 0.05 follows the null hypothesis, i.e. the data sample belongs to a normal distribution, and if *p*-value < 0.05, it does not follow a normal distribution. To the notice, the obtained value of significance came out to be greater than 0.05, i.e., 0.282 (NLMR), 0.33 (SL), and 0.057 (UC). The *p*-values for every smell are stated in Table 5.

#### Robust statistics

The battery usage evaluation for each version of every application has been carried out as a manual estimation and possibly may have outliers. Thus, ’Maximum likelihood Estimation’ (M- M-estimation) was applied to help identify outliers or extreme observations and estimate the value of battery consumption based on observed data. However, M-estimators appear to dominate the field due to their generality, high breakdown point, and efficiency^12^. The influence function of an M-estimator is proportional to $\psi$ (explained in further section 4.2.2), inferring that the properties of such an estimator like rejection point, gross-error sensitivity or local – shift sensitivity can be derived^12^.

#### Choice of $\psi$:

The choice of the $\psi$ function is not critical to gaining a good robust estimate, and many choices will give similar results that offer great improvements, in terms of efficiency and bias, over classical estimates in the presence of outliers.

The most used robust estimators are Huber’s M-estimator, Hampel estimator, and Tukey’s biweight estimator. After studying various selection functions, Tukey’s biweight function is selected as a study recommends the biweight function with the efficiency at the normal set to 85% theoretically^32^. The biweight correlation is calculated as follows:

1 $$\begin{aligned} R_{ij}= S_{ij}/(\sqrt{(}S_{ii}S_{jj})) \end{aligned}$$

The objective function of biweight is:

2 $$\begin{aligned} \rho \left( x\right) ={\ \frac{1}{6}[1-({1-u^2)}^3]} \end{aligned}$$

And $$\psi$$ - function,

3 $$\begin{aligned} \psi (u) = {u({1-u^{2\ })}^2} \end{aligned}$$

The weighing constant of Tukey Biweight is 4.685. With Tukey’s biweight function, the analysis of data values estimated as the change between the smelly and refactored version is taken as the sample set. The maximum likelihood value is obtained, and robust statistics is applied to determine the range in which the distribution of population lies with a 95% confidence level. The lower bound and the upper bound of the 95% confidence level, along with the skewness and kurtosis, is described in Table 6.

Tukey’s biweight^16^ analysis produced a maximum likelihood value for each smell, which led to a battery percentage conservation in each smell after refactoring. This result ensures that, depending on the functionality of the application, the refactoring process will undoubtedly reduce battery usage by a specific percentage.

**Table 6:** Robust statistics data of Tukey biweight function for NLMR, SL, and UC smell.

Robust Statistics Attributes	NLMR	SL	UC
(Note: 95% Confidence Level)			
Lower Bound	22.58 × 10^3^	18.05 × 10^3^	20.5 × 10^3^
Upper Bound	73.42 × 10^3^	51.95 × 10^3^	71.5 × 10^3^
Mean	48 × 10^3^	35 × 10^3^	46 × 10^3^*
Standard Dev.	35.528	23.688	35.653
Skewness	0.967	0.627	1.164
Kurtosis	0.445	$-0.342$	0.494

**Figure 4:**
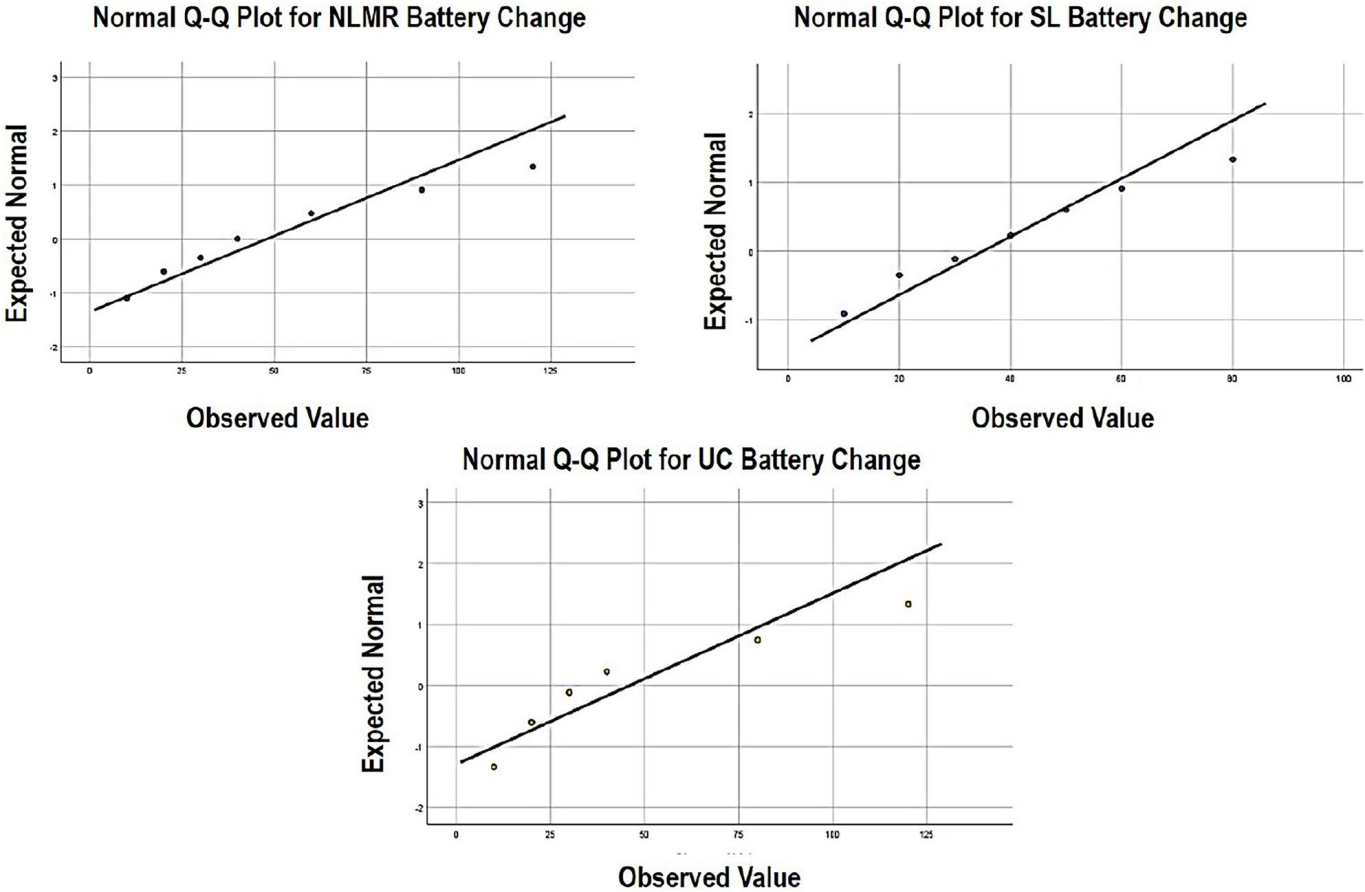
Q–Q plot for the expected normal and observed values for considered code smells.

The expected values obtained using M-estimation and the observed values of battery consumption of both versions of all the applications have been graphically plotted using the Q–Q plots in Fig. 4 for all the smells.

**Figure 5:**
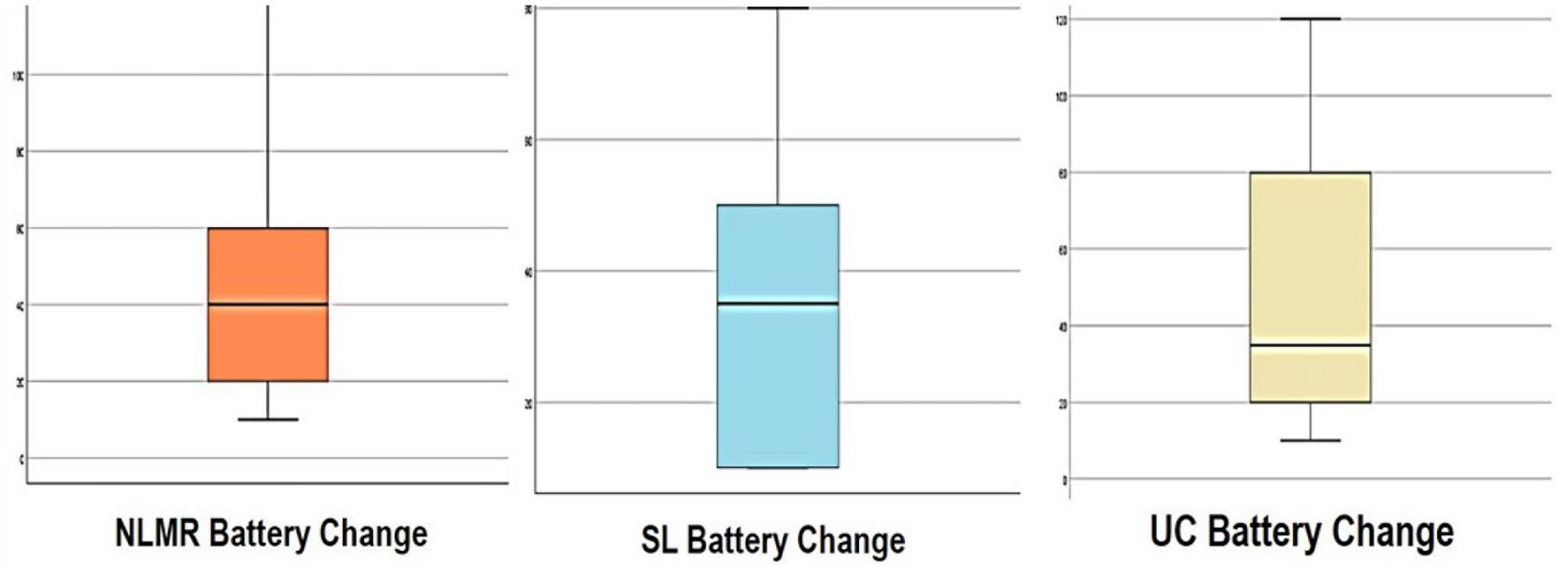
Box plot for the battery percentage change for considered code smells.

Here, the Q–Q plot represents a linear relationship between the distribution of expected and observed values of battery consumption. Figure 4 shows the Q–Q plots for the expected normal and observed values for NLMR, SL, and UC. Figure 5 shows the box plot graphs referring to the battery percentage changes in the respective smells-the box plot summarizes numerical data groups through their quarterlies.

This highlights that refactoring code smells in Android helps in energy conservation and motivates us to build a battery consumption model. Thus, the effect of refactoring on battery conservation for ten applications for each smell by Tukey Biweight M- estimation is answering RQ2. These values are represented as the maximum likelihood value, examined independently for each smell. Hence, it can be inferred that, through the analysis of 10 applications out of 16, some percentage of battery is preserved when a smell is refactored. So, the summary for all three code smells is given below:

For NLMR code smell, 38.4 × 10^−3^% battery is conserved.

For SL code smell, 33.36 × 10^−3^% is conserved.

For UC code smell, 29.57 × 10^−3^% is conserved.

### RQ3: Can a mathematical model be derived for estimating battery usage through software code metrics of smelly code?

RQ3 aims to devise a relationship between the battery data obtained from the manual real-time-based running of smelly and refactored applications and the changes in the applications observed through the static code metrics.

#### Pre-modelling phase

As discussed in section 4.1, RQ1, a method for refactoring process to reduce the amount of battery consumption in real-time scenarios has been discussed. Similarly, we need to scrutinize a static code analyzer tool (Scitools Understand, as mentioned in previous sections) to determine whether the source code metrics reflect the changes between the refactored code and the smelly code for each smell. The tool analyzed each code, returning up to 40 metrics (https://www.meteonic.com/understand) for Java files at the class level for all three smells (NLMR, SL, UC) individually for the 10 applications out of 16. While investigating the two datasets for each smell, the refactoring involves changes in the static code metrics. These changes have been observed for the majority of the data at the class level. Table 7 shows the percentage change for metrics inspected for NLMR, SL, and UC smells, respectively.

**Table 7:** Metric changes quantitative analysis.

Quantitative Analysis Factors for Metric Changes	NLMR	SL	UC
No. of classes compared (Smelly class which were further refactored)	1147	1424	4452
Classes containing mismatches (Classes whose metrics reflect changes before and after refactoring)	108	248	96
% Changes	9.4%	17.4%	2.2%

After working with RQ1 and RQ2 as in sections 4.1 and 4.2, respectively, we can state the following two facts.

D1: Refactoring smells caused significant changes in static metrics in a percentage of classes, as shown in Table 7. It is worth noticing that NLMR and SL reflected a relevant number of changes; however, UC smell reflects only 2% of the total change.

D2: The smelly and the refactored version of each application showed differences in battery usage values (tested with the Tukey biweight test) during the 10-minute run. The refactoring process implies a significant change in battery consumption, which was statistically altered to minimize the error while estimating the battery levels through Batterystats. The maximum likelihood value was 38.4 × 10^−3^% (NLMR), 33.36 × 10^−3^% (SL), and 29.57 × 10^−3^% (UC) as obtained in RQ2.

These two facts suggest that the changes in static code metrics can also impact the battery consumption levels of Android smartphones. This motivates us to explore a relationship between the above two facts using a multi-linear regression model. Considering the maximum likelihood value, a calculated refactored value was obtained to remove the inequality (as discussed in D2) and gain consistency in the results to create the best-fit model for predicting the battery usage:

*Calculated Refactored Battery Value = Observed Smelly Battery Value − Maximum Likelihood Value*

#### Multi-linear regression analysis

The independent variables in the multi-linear regression model emerged from the metrics for both the versions (smelly and refactored) of all the package-level applications. The dependent variable will be the battery usage of the concerned package. As battery historian data are linked to packages, code metrics at the package level are considered for the study. Further, multi-linear regression is applied for each code smell, where we have divided the dataset of 10 applications into training (70%) and testing (30%).

**NLMR regression model:** It is preferred as there should be collinearity between the independent and the dependent variable for successful regression analysis. However, there should not be multicollinearity between the independent variables themselves. There are two independent variables (CountLineCodeExe, CountDeclMethodPublic) used for modeling the NLMR smell. A total number of 24 metrics reflected changes after refactoring CountLineCodeExe and CountDeclMethodPublic with 7.6 % and 7.0% of changes in metric values were selected. This percentage accounts for 87 and 80 classes, respectively. These metrics were not too highly correlated with each other. The correlation coefficient was 0.606, and the significance value was 0.005 (Two-tailed Pearson significance).

1. Correlation matrix: The correlation coefficients among the independent variables were 0.606, which is smaller than 1 (significant) using Pearson bi-variate correlation.
2. Tolerance (T): It regulates one independent variable’s influence on other independent variables. T<0.1 is considered almost a perfect linear combination of the independent variables. The tolerance factor was:
  CountLineCodeExe: 0.632 and CountDecl-MethodPublic: 0.632.
3. Variance Inflation Factor (VIF): It is defined as the reciprocal of the tolerance value. If VIF > 5, it indicates the slight presence of multicollinearity, whereas if VIF > 10, 3 multicollinearity certainly exists among the variables. The VIF for NLMR modeling is described as CountLineCodeExe: 1.582 and CountDecl-MethodPublic: 1.582. The information extracted from the NLMR multi-linear regression was employed to generate the equation of battery consumption affected by software metrics. The equation obtained from the multi-linear regression model for NLMR is:
  4 $$\begin{aligned} Y = 0.893 \times x1 + (-0.052) \times x2 + \beta \end{aligned}$$
  where
  x1 = CountDeclMethodPublic
  x2= CountLineCodeExe
  $$\beta$$ = 107.718
  Y = Battery consumption ($$10^3$$)
  By obtaining the significance value for the correlation matrix, tolerance, and the VIF value, the model designed gives the following information: R = 0.872, which describes the relationship between the dependent and the independent variable, and R-Square = 0.76, a coefficient of determination of how close the data are to the fitted regression line as described in Table 8.
  All the variables and constants used in the model are significant, as shown in Table 9.

**Figure 6:**
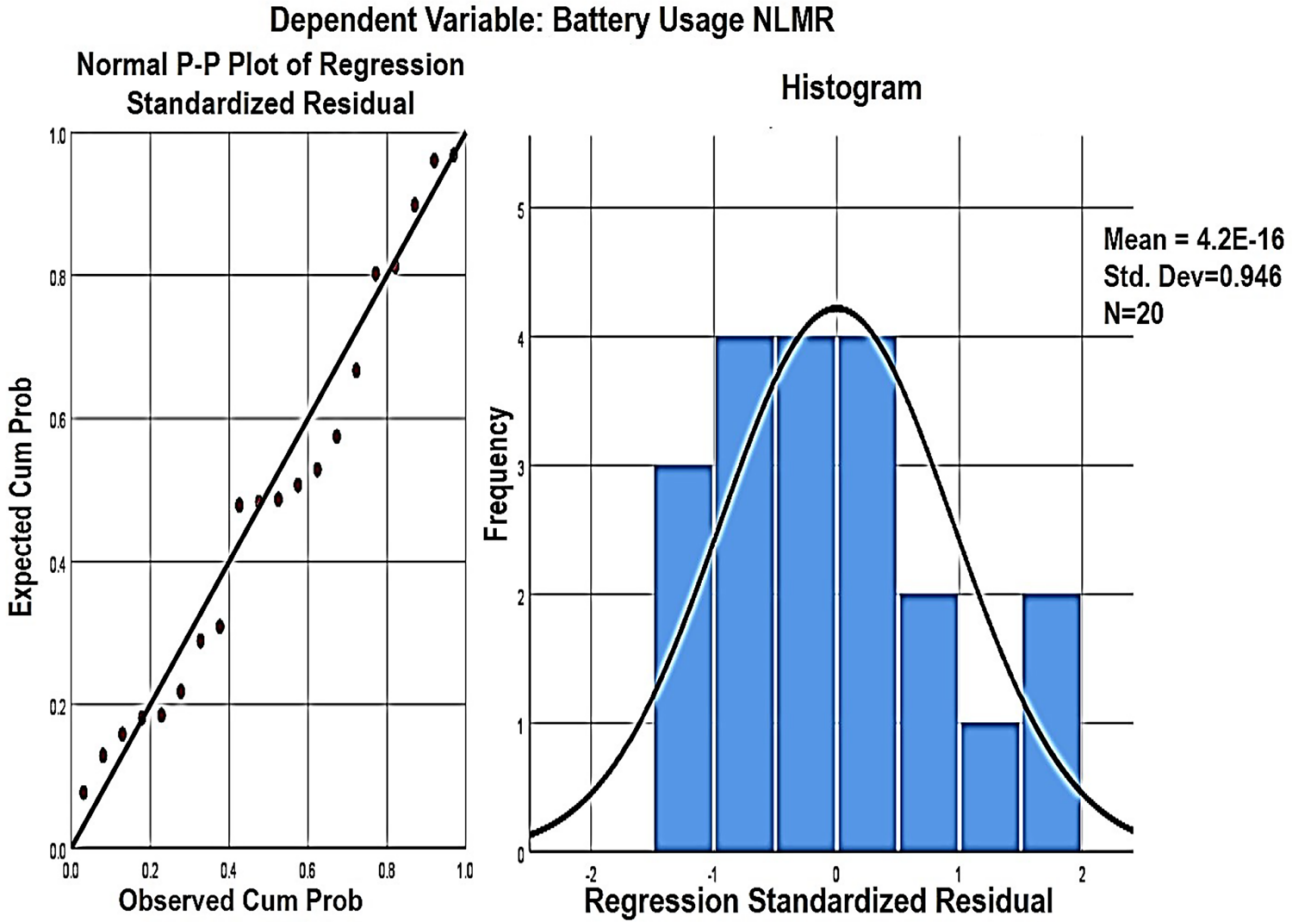
Normal P–P plot and regression standardized residual plot for NLMR smell.

The graphical representation of regression residuals and normal p–p plot of the regression residual is described in Fig. 6. The p–p plot compares the expected cumulative distribution function (CDF) of the normal distribution to the observed CDF of the standard residual. The standardized regression residuals represent the measure of the strength of the difference between observed and expected values.

**Table 8:** Proposed model summary for NLMR code smell.

NLMR model summary	Values
R	0.872
R-Square	0.76
Adjusted R-square	0.732
Standard error of the estimate	42.2809
R-square change	0.76
F change	26.924
df1	2
df2	17
Sig. F change	0

**Table 9:** NLMR code smell coefficient with their statistics.

Independent variables	Unstandardized coeff.		Standardized coeff.				
	Beta	Std. error	Beta	t	Significance	Tolerance	VIF
(Constant)	107.718	15.425		6.983	0		
CountDeclMethodPublic	0.893	0.123	1.086	7.269	0	0.632	1.582
CountLineCodeExe	$-0.052$	0.01	$-0.778$	$-5.206$	0	0.632	1.582

**SL regression model:** The same process done for NLMR is repeated for SL smell. A total number of 32 metrics reflected changes after refactoring out of which CountLineCode and CountDeclInstanceVariable metrics with 10.5% and 7.3% changes respectively were selected. This percentage accounts for 149 and 104 classes, respectively. The independent variables i.e., the metrics considered here are CountLineCode (10.5%) and CountDeclInstanceVariable (7.3%). They represent a substantial correlation of 0.424. The dependent variable, on the other hand, the battery usage data, has a good correlation with the metrics used. The multi-collinearity criteria obtained for SL:

1. Correlation Matrix: Pearson’s Bivariate correlation – 0.424 (two-tailed) .
2. Tolerance (T): CountLineCode:0.357 and CountDeclInstanceVariable:0.357.
3. Variance Inflation Factor (VIF): CountLineCode:2.8 and CountDeclInstanceVariable:2.8.

The final model obtained from the above analysis is stated below:

5 $$\begin{aligned} Y = 2.13\times z1 + (-0.038)\times z2 + \alpha \end{aligned}$$

where

- z1 = CountDeclInstanceVariable
- z2= CountLineCode
- $$\alpha$$ = 111.665
- Y = Battery consumption ($$10^3$$)

**Figure 7:**
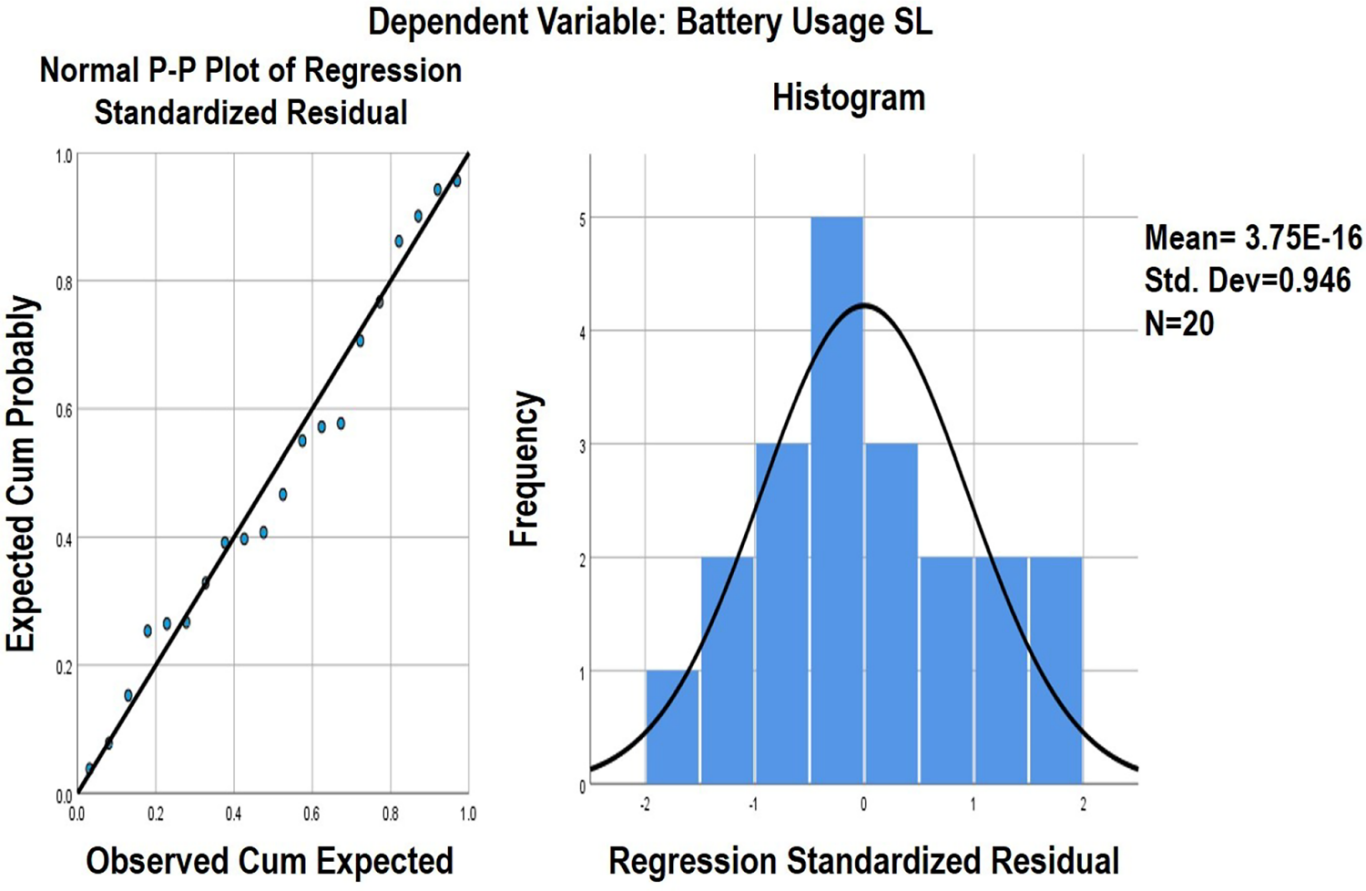
Normal P–P plot and regression standardized residual plot for SL smell.

By obtaining the significance value for the correlation matrix, tolerance, and the VIF value, the model designed gives the following information: R = 0.817 represents the relationship between the independent variable and the dependent variable, and R-Square = 0.668, a coefficient of determination of how close the data are to the fitted regression line as described in Table 10. All the variables and constants used in the model are significant, as depicted in Table 11. The graphical representation of regression residuals and normal p–p plot of regression residuals for SL smell are described in Fig. 7.

Unlike NLMR and SL, which reflect notable changes, UC smell reflects only 2% of the total metric composition change analysis. So, regression modelling has not been performed on UC smell as it is considered irrelevant for battery consumption.

**Table 10:** Summary of proposed model for SL code smell.

SL Model Summary	Values
R	0.817
R-square	0.668
Adjusted R-square	0.628
Standard error of the estimate	92.38859
R-square change	0.668
F change	17.067
df1	2
df2	17
Sig. F change	0

**Table 11:** SL code smell coefficient with their statistics.

Independent variables	Unstandardized coeff.		Standardized coeff.				
	Beta	Std. error	Beta	t	Significance	Tolerance	VIF
(Constant)	111.665	37.439		2.983	0.008		
CountLineCode	$-0.038$	0.017	$-0.534$	$-2.28$	0.036	0.357	2.8
CountDeclInstanceVariable	2.13	0.422	1.18	5.043	0	0.357	2.8

Therefore, we have proposed two models for their respective smells, i.e., NLMR and SL, stating that the larger the R-square value, the better the regression model fits the predicted value in RQ3.

### RQ4: How does the metric distribution affecting the Android application’s battery consumption correlate to the measured distribution associated with refactoring rules?

The goal linked to RQ4 is to describe the correlation between the metric distribution affecting battery consumption and the metric distribution associated with refactoring rules. As in section 4.3, RQ3, we found a relationship between battery consumption and metrics. In RQ1, each smell is tested for refactoring rules from some selected metrics; these two statements suggest a strong association between the two types of metric distributions. We explored the correlations between the metrics for the 3 smells: SL, NLMR, and UC.

Table 7 shows that there is a small variation between smelly and refactored versions with respect to software metrics. Hence, a linear model for UC smell could not be devised.

The observation of the data for metric distribution revealed a monotonic relationship between the metrics affecting battery consumption and metrics associated with refactoring rules. We have used Spearman’s correlation to test the degree of a monotonic relationship between the metric distributions. The Spearman rank-order correlation coefficient is a non-parametric measure of the direction of the association and the strength that exists between two variables measured on at least an ordinal scale. In a sample, it is denoted as follows:

6 $$\begin{aligned} -1\ \le \ \rho \ \le \ 1 \end{aligned}$$

It is worth noting that, unlike Pearson’s correlation, there is no requirement for normality of data, and hence, it is a non-parametric statistic.

The following formula is used to calculate the Spearman rank correlation:

7 $$\begin{aligned} \rho =1-\frac{6\sum d_i^2}{n\left( n^2-1\right) } \end{aligned}$$

where

- $\rho$ = Spearman rank correlation
- $d_i$ = the difference between the ranks of corresponding variables
- *n* = number of observations

A correlation value that is closer to +1 or $-1$ depicts a strong positive or a negative relationship, respectively.

**Table 12:** Spearsman correlation between replicated rule metric and metric affecting battery for NLMR smell.

Battery model metric (Note: Correlation Coefficient values are calculated at .01 level significance)			
Ref.Method Metric		CountDeclMethodPublic	CountLineCode
MaxInheritanceTree	Correlation Coefficient	.532	$-.098$
	Sig. (2-tailed)	0.000	0.001
PercentLackOfCohesion	Correlation Coefficient	.621	.526
	Sig. (2-tailed)	0.000	0.000
CountStmtExe	Correlation Coefficient	.857	.427
	Sig. (2-tailed)	0.000	0.000
	N	1147	1147

A correlation value closer to +1 or $-1$ depicts a strong positive or negative relationship respectively.

The following results appear from the NLMR dataset’s estimation: CountStmtExe depicts a strong relation of 0.857 with CountDeclMethodPublic. Subsequently, PercetLackOfCohesion depicts a strong relation of 0.628 with CountDeclMethodPublic, as shown in Table 12.

From the estimation of the SL dataset: it shows a strong relation of 0.857 with CountDeclMethodPublic, CountLine shows a strong relation of 0.989 with CountLineCode, CountStmtExe shows a strong relation of 0.615 with CountDeclInstanceVariable as shown in Table 13. The following results appear from the estimation of UC dataset: SumCyclomaticModified shows a firm relation of 0.84 with CountStmtExe, and MaxInheritanceTree shows a strong relation of 0.722 with CountClassCoupled, as shown in Table 14. Thus, the metric distribution affecting the Android application’s battery consumption highly correlates to the metric distribution associated with refactoring rules. This infers a strong relationship between the refactoring rules and the battery usage model.

**Table 13:** Spearsman correlation between replicated rule metric and metric affecting battery for SL smell.

Ref replica metric		Battery usage metric (Note: Correlation Coefficient values are calculated at .01 level significance)	
		CountLineCode	CountDeclInstanceVariable
CountStmtExe	Correlation Coefficient	.329	.615
	Sig. (2-tailed)	0.000	0.000
CountLine	Correlation Coefficient	.989	.461
	Sig. (2-tailed)	0.000	0.000
	N	1424	1424

The following results were obtained from the estimation of the SL dataset. shows a strong relationship of 0.857 with CountDeclMethodPublic CountLine shows a strong relationship of 0.989 with CountLineode CountStmtExe shows a strong relationship of 0.615 with CountDeclInstanceVariable

**Table 14:** Spearsman correlation between replicated rule metric and metric affecting battery for UC smell.

UC ref replica metric		UC ref changed metric (Note: Correlation Coefficient values are calculated at .01 level significance)		
		MaxNesting	CountStmtExe	CountClassCoupled
MaxInheritanceTree	Correlation Coefficient	.425	.713	.722
	Sig. (2-tailed)	0.000	0.000	0.000
CountLineCodeDecl	Correlation Coefficient	$-0.002$	$-.215$	$-.260$
	Sig. (2-tailed)	0.916	0.000	0.000
CountStmtDecl	Correlation Coefficient	0.001	$-.204$	$-.243$
	Sig. (2-tailed)	0.921	0.000	0.000
SumCyclomaticModified	Correlation Coefficient	.758	.840	.700
	Sig. (2-tailed)	0.000	0.000	0.000
CountStmt	Correlation Coefficient	.234	.087	0.022
	Sig. (2-tailed)	0.000	0.000	0.133
	N	4452	4452	4452

**Table 15:** A Comparison between closely related existing literature with presented research.

	Android Smells observation with mobile applications	Energy Consumption of mobile apps	Maths model	S/W metrics distribution changes with Refactoring	Correlation for S/W metrics and Battery Consumption	Apps for Industry
Presented Study	Yes	Yes	Yes	Yes	Yes	Yes
Palomba et al.^24^	Yes	Yes	No	No	No	Yes
Pereira et al.^73^	Yes	Yes	No	No	No	Yes

A comparative analysis detailing the key differences between the proposed study and the previously published research articles that are closely related can be reviewed in the Table 15. In this table two referenced research articles are chosen, highlighting their similarities and differences in terms of research focus and methodology. The presented research differs from previous publications mostly due to the significant contributions made to the mathematical model and correlation factor between software metrics and battery consumption.

## Threats to validity

In the context of this study, code smell analysis is performed using the ’aDoctor’ tool proposed by Palomba et al.^23^. The ’aDoctor’ tool detects the absence or the presence of the smells at the class level but not the amount of presence of code smells in a class. Also, we can’t overlook the fact that ’aDoctor’ excludes some smells and that other existing tools have different threshold values for bad smells, which could impact the findings on bad smells. Only the Android software data set is allowed by the model shown here.

The term “external validity” refers to how generalized a conclusion represents. Only Android software is allowed by the model presented here. The model must also be implemented for additional languages in order for the results to be generalized.

Although a regression model is proposed for mobile battery usage through software code metrics of smelly code, different statistical methodologies can lead in a different direction.

Moreover, NLMR, SL, and UC’s refactoring as a manual process might escalate the code’s complexity. In addition, the manual battery analysis might indicate a human error while running the smelly and the refactored applications in terms of performed actions.

Furthermore, Battery Historian only provides a rough approximation of the energy consumption and only roughly attributes this to the app. Therefore, there is a measurement error introduced by this methodology.

Other real time challenges that we have faced at the time of implementation are resource utilization, CPU stress, battery health, mobile use, and the temperature impact on hardware and battery, may impact the battery consumption. However, this study focuses on code smells, which is one of the crucial factors in the software development process.

## Conclusion and future scope

This research aims to analyze the prominent consequences of the Android code smells on the power consumption by Android applications on smartphones. The Android applications have been fixed from code smells through refactoring methods. Moreover, it has also been observed that the software metrics of Android applications play a vital role in inspecting them for refactoring. Furthermore, the difference in battery consumption observed between the smelly and the refactored applications verifies the positive impact on energy preservation. The conserved energy can enhance the battery life and can be reused for other operations, thus promoting an approach to green energy.

This work has also been capable of devising a mathematical model for the three considered smells, which might help developers estimate the amount of battery usage before deploying the application. This technique would surely help Android Java developers optimize performance at the application level and build efficient, time-saving, optimized code even from the initial phases of the development cycle.

This research analyses the impact of three Android-specific code smells, namely NLMR, SL, and UC, which the developers may easily ignore and can hamper Android smartphones’ performance. The research involved the refactoring of 16 applications containing smells with 4166 classes, which were also manually analyzed for battery consumption. We statistically evaluated the percentage composition of battery usage of smells using maximum likelihood values, which helped in maintaining the consistency of data for linear regression model prediction. The data analysis enabled us to reach the following conclusions:

Conclusion 1: The analysis of software code metrics when refactoring code smells enabled a new approach through refactoring rules derived from rule-based machine learning classifiers. This approach reached a prominent precision against the JRip rule-based classifier with values of 80.8%, 86.4%, and 90.3% for NLMR, SL, and UC, respectively.

Conclusion 2: The refactoring of considered code smells also ensured a comparative decrease in battery consumption when tested at the package level. This was statistically corrected through the maximum likelihood value, expressed as the expected refactored battery value. These values represented the battery conservation for the considered smells: 38.4 × 10^−3^% (NLMR), 33.36 × 10^−3^% (SL), 29.57 × 10^−3^% (UC). The estimated value of battery load and the software metrics of each version of applications at the package level were then tested for linear regression modelling and drafted as an equation only for NLMR and SL. However, UC was not considered for modelling due to its minor observed significant change between the smelly and the refactored version of all the applications. NLMR and SL were modelled at a good significance rate with all the factors (Tolerance and Variance Inflation Factor) mentioned in RQ3 (NLMR and SL) modelling. These two models (NLMR and SL) can then be used to check the battery estimations and provide the concerned software metric values. The R-Square value for the NLMR model accounts for 76% fitness, whereas SL is 66.8%.

Conclusion 3: It was possible to devise a relationship between the metrics impacted by the refactoring rules (as proposed in RQ1) and the metric distribution of models (as proposed in RQ3) for each smell using Spearman correlation. This leads to the verification of a strong relationship between the proposed refactoring method and the battery-metric model that affects the Android applications. The three smells showed a significant correlation between the respective metrics of the battery model and the refactored rules, stated in Table 11,12,13.

The above-stated conclusions can be further analyzed in the future for different performance measures with different smells impacting them. In accordance with the research that was presented, Android code smells affect how long a phone’s battery lasts. Only three smells have been taken into consideration here; other smells can be examined for improved battery saving.Also, the battery estimations can be analyzed for various other smells of other commonly used languages. Furthermore, this analysis can be carried out on desktop applications to enhance the energy performance of personal desktop computers, reduce the battery consumption of portable computers, and improve software quality and maintainability. This work can be extended to include the parameters related to the battery. We also plan to validate the model on the other applications with different programming languages software as well. There is still opportunity to conduct more research on iOS-enabled phones, especially in consideration of the pervasive problem of high battery consumption in modern world.

## Data Availability

The data is available at* https://bitbucket.org/sumit3sep/raw_data/src/master/ *Please ensure an underscore between raw and data while using this link online. Sometimes, the underscore disappears when clicking the link.
